# Barcoding Life's Matrix: Translating Biodiversity Genomics into High School Settings to Enhance Life Science Education

**DOI:** 10.1371/journal.pbio.1001471

**Published:** 2013-01-29

**Authors:** Linda Santschi, Robert H. Hanner, Sujeevan Ratnasingham, Michelle Riconscente, Ralph Imondi

**Affiliations:** 1 Integrative Biosciences Program, Coastal Marine Biolabs, Ventura Harbor, California, United States of America; 2 Biodiversity Institute of Ontario, University of Guelph, Guelph, Ontario, Canada; 3 Department of Integrative Biology, University of Guelph, Guelph, Ontario, Canada; 4 New York Hall of Science, Queens, New York, United States of America

## Abstract

From biomes to genomes: Innovating life science education by engaging students as citizen scientists in the International Barcode of Life project.

## Introduction

A comprehensive review conducted by the US National Academies' National Research Council suggests that most high school laboratory experiences fail to conform to established guidelines for effective science instruction [1]. These guidelines advocate designing laboratory experiences such that they produce clear and discernible outcomes, merge science content with learning about the *process* of science, and integrate hands-on activities into a sequence of traditional didactic course instruction. In addition to these basic guidelines, proponents of science education reform encourage more open-ended instructional strategies that bridge science research and education, and engage students in the use of modern tools of scientific inquiry [2],[3].

For high school science teachers, meeting these guidelines and recommendations may be especially challenging in the domain of molecular life science and bioinformatics. The rapid growth of this field, together with the inherently complex, abstract, and interdisciplinary nature of its scientific content, presents a number of profound educational challenges for teachers [4]. These challenges include (1) selecting appropriate content to disseminate, (2) defining an appropriate level of detail/depth of concepts and processes for students to understand, (3) integrating knowledge and methods across allied scientific fields, (4) emphasizing functions over facts, and (5) modeling how scientific information is generated and disseminated in real-world settings [4]. In light of these fundamental obstacles and more practical issues associated with teacher preparation, overcrowded classrooms, aggressive testing regimes, ill-equipped science labs, and limited access to scientific literature and information [1], it may be unrealistic to expect teachers operating within public high school learning settings to effectively manage purely open-ended forms of research-based inquiry in this rapidly evolving domain.

Discovery-based science education represents a structured alternative to open-ended forms of hands-on inquiry that is now being employed in a number of secondary and post-secondary settings to address science education reform agendas [5]–[10]. In the context of molecular life science education, this particular form of instruction links domain knowledge, laboratory methods, and bioinformatics (or computational biology) within the framework of a complete and integrated analytic workflow that culminates in a tangible scientific output and a bona fide contribution to a particular body of scientific knowledge. From our perspective, the structure imposed by a discovery-based mode of instruction is particularly well suited to high school learning settings because it helps teachers overcome some of the more challenging pedagogical aspects of molecular life science education outlined above [4]. Furthermore, discovery-based instruction actively promotes ongoing student reflection and discussion, and effectively models how scientific information is generated to formulate new hypotheses for the advancement of scientific knowledge. A carefully conceived model should therefore provide students with a priori knowledge of the types of research questions that their work can help address, and present novel opportunities for them to formulate and test hypotheses derived from the scientific information generated by their predecessors, thereby disambiguating the discovery–investigation continuum for young learners.

DNA barcoding [11] is a particularly suitable platform for molecular life science education that articulates a clear path from discovery-based science to novel investigational studies. This new system of eukaryotic species identification links metadata associated with a retrievable and taxonomically verified morphological voucher specimen to corresponding nucleotide sequence (barcode) data obtained from gene loci that delimit species boundaries: the mitochondrial *cytochrome c oxidase subunit 1 (CO1)* gene for animals [11], the chloroplast *ribulose-1,5-bisphosphate carboxylase (rbcL)* and *maturase K (matK)* genes for land plants [12], and the nuclear ribosomal internal transcribed spacer (ITS) region for fungi [13]. Reference DNA barcode records containing morphological, geospatial, and molecular genetic data reside within the Barcode of Life Data Systems (BOLD), an online public-access analytical workbench and data repository [14]. A multinational alliance of scientists and institutions working under the International Barcode of Life (iBOL) project are currently populating BOLD with reference barcode records for the species with the highest socioeconomic importance to humanity. The information instilled in reference barcode records by this global community enables BOLD end-users to taxonomically identify unknown or unidentifiable specimens with barcode sequence data alone (by querying sequence data derived from an unknown or unidentifiable specimen against complete reference barcode records contained in the BOLD data repository). BOLD currently contains approximately 1.8 million barcode records representing 160,000 species of plants, animals, and fungi. This information is already being used to address a variety of applied problems, including the detection of market substitution [15] and food adulteration [16], the illegal trade of endangered species [17],[18], the arrival of harmful agricultural pests [19],[20], and the presence of disease vectors [21],[22].

The Barcoding Life's Matrix program was launched to enlist the participation of high school students in iBOL as citizen scientists. The project consists of two interrelated research phases: (1) a discovery phase that engages students in the creation of a well-parameterized reference barcode library for a targeted list of marine taxa, and (2) an investigational phase in which students utilize this data collection to conduct novel, authentic, and regionally relevant ecological studies in collaboration with our scientific partners. Here, we briefly summarize the nature and scope of the project, its primary educational components, and the results generated by its student participants. We also announce the availability of new mobile computing and web-based technologies to broaden the involvement of secondary and post-secondary students in the DNA barcoding enterprise (more detailed information can be found online at http://www.studentDNAbarcoding.org).

## Engagement Strategies

The project adopts two primary strategies for student engagement: residential research experiences for 11th- and 12th-grade high school students, and professional development workshops for high school science teachers. During immersive, seven-day residential research institutes hosted at Coastal Marine Biolabs (the lead organization for this project), students work alongside scientists as they conduct interrelated field, laboratory, and bioinformatics activities that culminate in the submission of professional quality reference barcode data to BOLD and the International Nucleotide Sequence Data Collaboration (INSDC). In addition to performing field and laboratory work, students prepare and present a synthesis of their residential research experience at public mini-symposia hosted at the Robert J. Lagomarsino Visitor Center of Channel Islands National Park. These presentations form part of larger public engagement events that include keynote lectures from visiting research scientists, conservation biologists, environmental policy makers and attorneys, and other members of the professional community.

The professional development project component provides high school science teachers with in-depth pedagogical and procedural training, multimedia instructional materials, and research-grade equipment needed to engage students in the generation and submission of reference barcode data within their own science laboratories. Enrollment in the professional development program requires a minimum 12-month commitment from teacher participants and an expression of support from a school- or district-level administrator. During each 12-month program cycle, teachers attend a comprehensive eight-day professional development workshop series, complete associated workshop evaluations, formulate and submit a formal student assessment plan, administer pre- and post-evaluation instruments to students, implement the project in a life science course, and participate in one or more learning community meetings to share their experiences and offer feedback for project improvement.

The program is supported by a comprehensive, standards-aligned curriculum that was carefully developed through extensive collaborations among scientists, educators, and bioinformaticians. For delivery in high school settings, the core curriculum associated with our residential research institutes was condensed into 16 instructional units that integrate concepts, processes, and principles from a diversity of life science disciplines that span taxonomy and systematics, ecology, environmental science, evolution, genetics, bioinformatics, and molecular, cellular, and developmental biology. To encourage in-school implementation, each instructional unit was specifically designed for delivery in a standard 50-minute class period. Multimedia instructional materials are deployed through an online content management system that is accessible on the teacher resource page of the Barcoding Life's Matrix website (http://studentdnabarcoding.org/resources/teacher-tools-and-support.html). Because the utilization and delivery of these resources form an important dimension of the overall project evaluation, educators are requested to create an account before viewing and presenting these materials to their students.

## Open-Access Technology Resources

**Figure 1:**
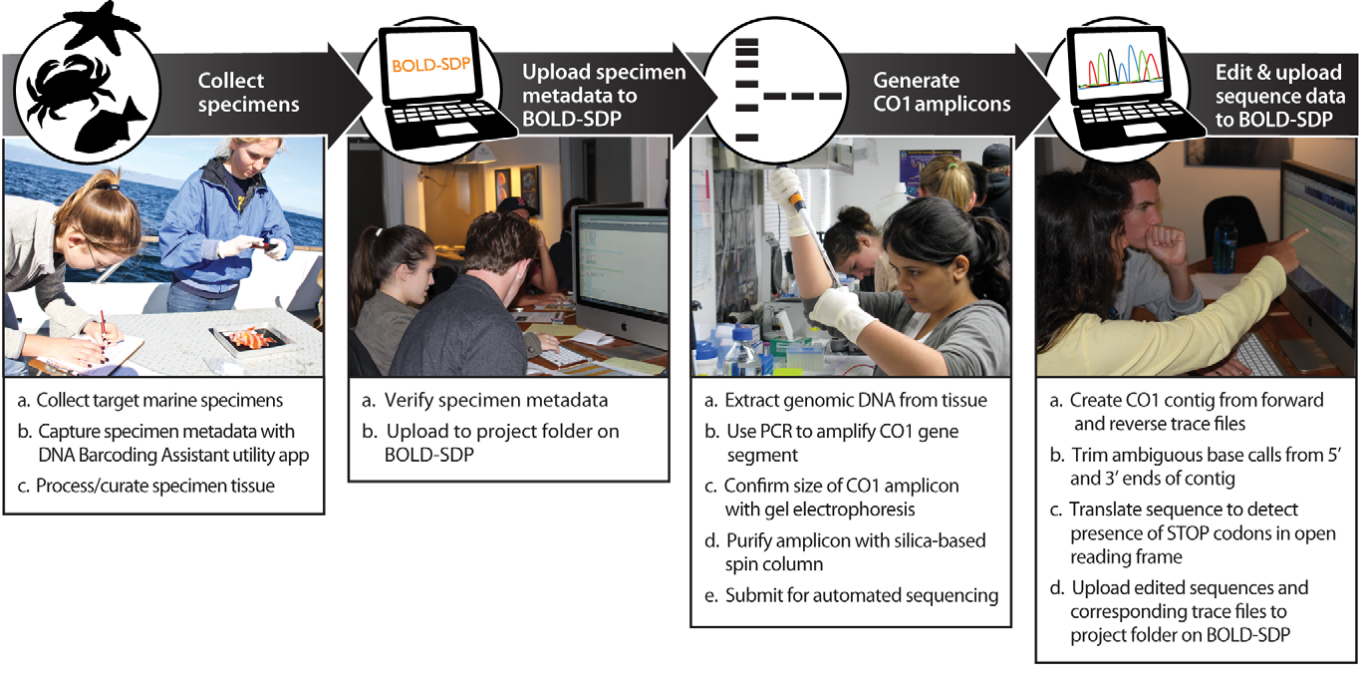
The DNA barcoding workflow. Students participating in the Barcoding Life's Matrix program are engaged in a complete and integrated analytic workflow that includes the following core activities: collecting targeted marine specimens and processing/curating corresponding tissue; recording specimen data and collection event details using mobile computing technology; uploading specimen data to BOLD-SDP, an online workbench and data repository that was specifically designed for the educational community (see Figure 3 for additional details); generating CO1 amplicons using accessible and widely applied molecular-biology-based laboratory techniques (e.g., gDNA extraction, PCR amplification, agarose gel electrophoresis, silica spin-column purification of PCR products); assembling contigs and editing nucleotide sequence data using a suite of Internet-based bioinformatics tools and software; and uploading raw trace files and edited nucleotide sequence data to BOLD-SDP.

**Figure 2:**
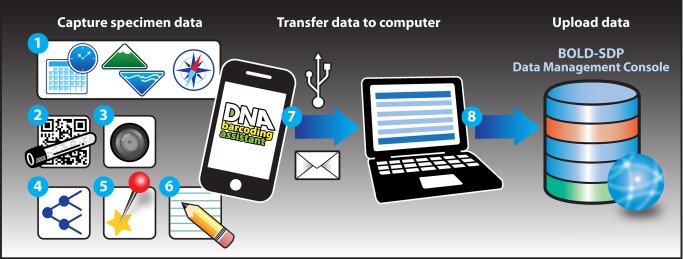
Acquisition and management of field data. Specimen data and collection event details are acquired by students in the field using the DNA Barcoding Assistant, a free smartphone utility application. The date and time, elevation/depth, and GPS coordinates are automatically captured by the app when a new specimen record is created (1). A unique specimen identifier can be entered into the record manually using the smartphone keypad, or by scanning a barcode symbol affixed to a specimen storage container using the barcode reader function of the app (2). Currently supported barcodes include DataMatrix, EAN/UPC product codes, QR codes, Code 128, Code 39, Code 93, DataBar (RSS), and Interleaved 2 of 5. A digital photo of the specimen is captured using the smartphone camera (3) and linked to the specimen record. Additional information, including provisional genus and species names (4), collection site designation (5), and field notes (including the name of the collector) can also be added to the record manually by using the smartphone keypad (6). Completed records are transferred from the app to a computer, directly or via email (7). Once data are inspected for accuracy, they can be submitted to BOLD-SDP through the student data management console (8).

**Figure 3:**
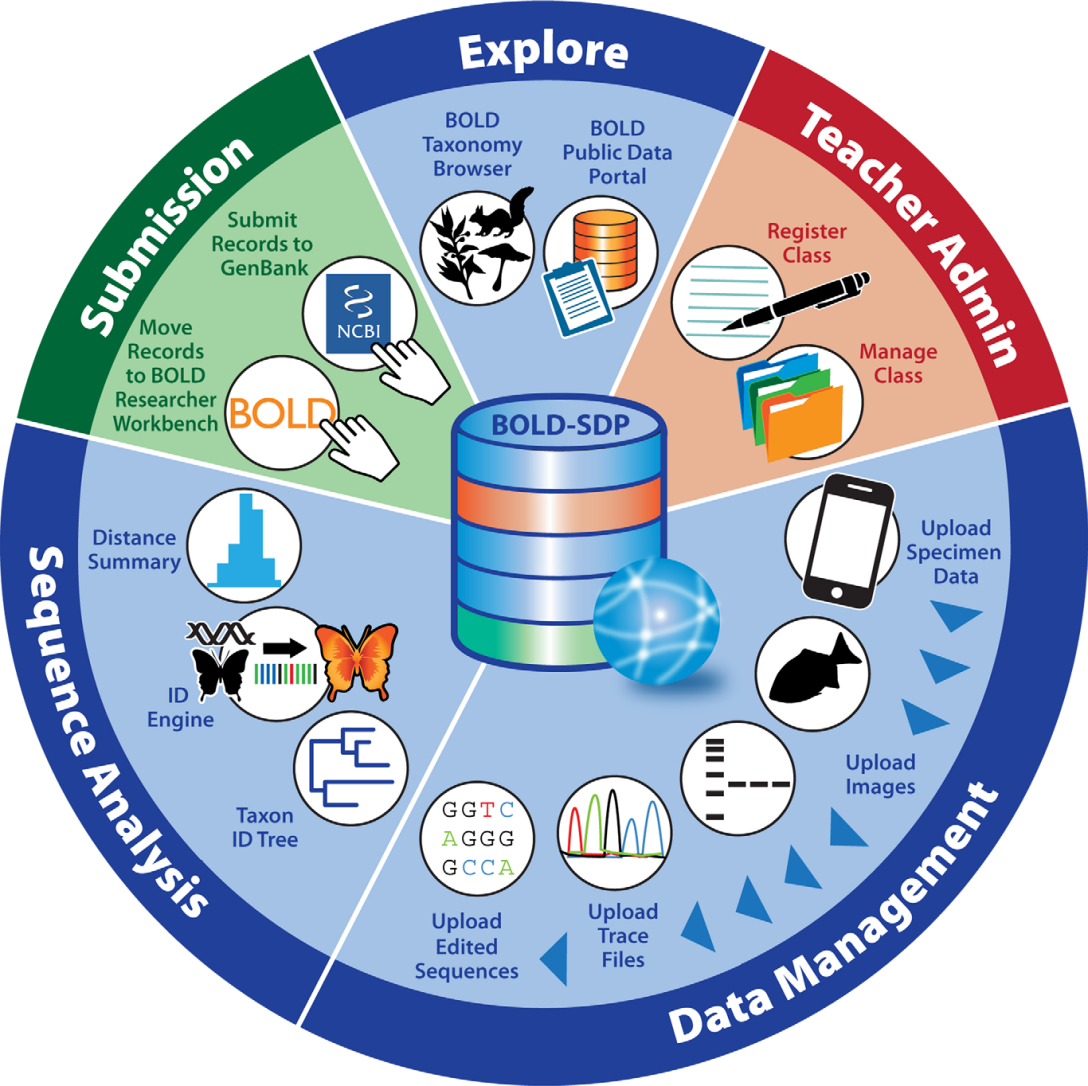
Barcode of Life Data Systems Student Data Portal (BOLD-SDP). BOLD-SDP is a public-access data repository, data management tool, and analytical workbench for educational users that consists of five customized consoles. The Explore console of BOLD-SDP provides students and teachers with a gateway to the BOLD Taxonomy Browser (where they can determine the barcoding status of specific taxa) and the BOLD Public Data Portal (where they can access and download data generated by professional iBOL scientists and researchers). Through this console, students and teachers can also access the Integrated Taxonomic Information System and US National Center for Biotechnology Information Taxonomy Database (where they can obtain the scientific name and accepted taxonomy of a specimen from its common name; not shown in figure). The Teacher Admin console was designed for educators to register their class, compile a roster of student contributors, create a destination folder for specimen and sequence data, monitor student progress, and inspect student-generated data for accuracy. The Data Management console of BOLD-SDP permits student users to upload specimen data and collection event details, images of specimens and lab results, forward and reverse trace files generated from their amplicons, and edited nucleotide sequence data. The Sequence Analysis console of BOLD-SDP contains an integrated suite of analytical tools that enables students to (1) visualize the relatedness of specimens by building a genetic distance-based phenogram or tree (Taxon ID Tree), (2) compare sequence data obtained from their specimens against barcode records contained in the BOLD data repository (to confirm their specimen identifications; ID Engine), and (3) calculate the differences among nucleotide sequences generated from their specimens (from species to class levels; Distance Summary). The Submission console of BOLD-SDP allows project leaders and members of the scientific community to vet student-generated barcode records before moving them to the BOLD researcher workbench and submitting corresponding nucleotide sequences for publication in GenBank/INSDC (refer to Figure 4 for additional details).

The hands-on segment of the project curriculum guides participants through the field, laboratory, and bioinformatics components of the barcoding pipeline by engaging them in the use of mobile computing technology, standardized molecular-biology-based laboratory techniques, and bioinformatics tools and databases (Figure 1). To preserve the evidentiary value of student-generated barcode data and facilitate compliance with quality standards established by the scientific community [14], an integrated suite of in silico resources was developed that help students acquire, manage, and analyze barcode data during different segments of the barcoding pipeline (Figures 2 and 3).

For instance, to standardize, streamline, and simplify data collection by students in the field, we developed the DNA Barcoding Assistant, a free smartphone utility application (Figure 2). The application provides an intuitive interface for student users to capture unique identifiers and compile records containing provisional user-assigned taxonomic identifications, time-stamped digital images, geospatial data, and collection event details for specimens obtained in the field (for more information, visit the support website at http://www.DNABarcodingAssistant.org).

**Figure 4:**
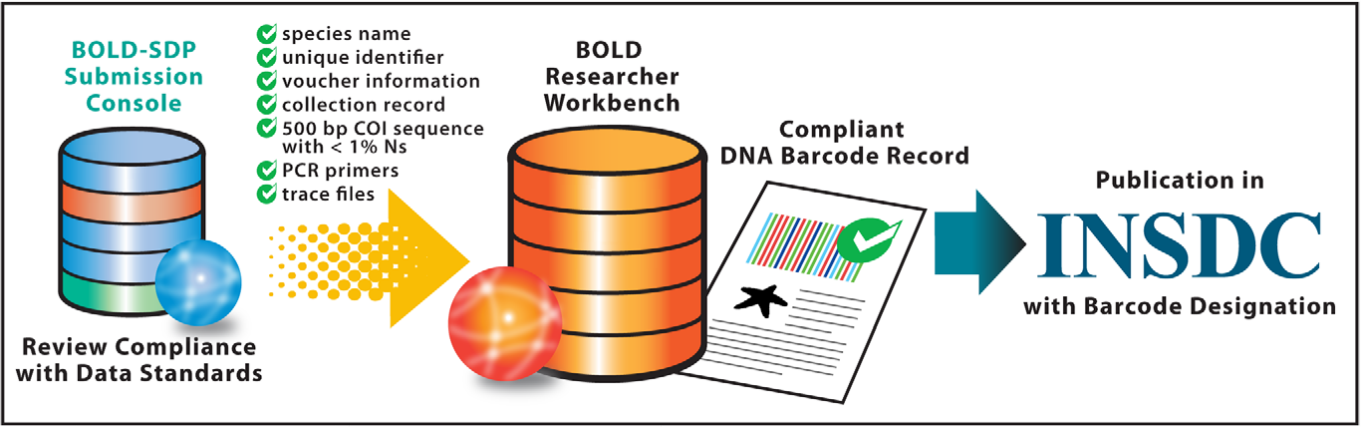
Meeting the Barcode Data Standard. Reference barcode sequences are linked to collateral data associated with the source specimen and collection event within biphasic records. Given their importance in ensuring the accuracy of species identifications through BOLD, reference barcode records are subject to a variety of formal data standards established by the scientific community. Through the Submission console of BOLD-SDP, project leaders and researchers review student-generated barcode records for their compliance with current data standards. Required data elements minimally include a species name assigned by an expert taxonomist (or a provisional name), a unique specimen identifier, information related to the voucher specimen (including the name of the institution storing the voucher), a collection record (e.g., collector, collection date, collection location, and geospatial coordinates), a CO1 sequence (for animals) of at least 500 nucleotides with fewer than 1% ambiguous base calls (Ns), the sequence of PCR primers used to generate the CO1 amplicon, and trace files. Student-generated records that satisfy these criteria are moved from BOLD-SDP to the BOLD researcher workbench and published in INSDC with the BARCODE designation.

We also developed the BOLD Student Data Portal (BOLD-SDP), a customized workspace and analytical workbench for educational users (Figure 3; http://www.boldsystems.org/edu). BOLD-SDP expands the suite of data management and analysis tools employed by the BOLD researcher workbench to perform additional sequence classification and tagging of barcode data generated by members of the educational community. Most of the data validation and visualization features of BOLD are exposed via web service to BOLD-SDP, and wrapped by audience-specific interfaces. This enables teachers and students using BOLD-SDP to utilize the same suite of tools used by professional scientists in the researcher workbench, but through simplified consoles that are more suitable for educational end-users. Following data validation by professional scientists (through the submission console of BOLD-SDP), student records are then moved to the BOLD researcher workbench and published in INSDC (Figure 4).

It bears noting here that educators often overlook the fundamental distinction between a barcode generated for the purpose of assigning a species name to an unknown tissue source or food product (“query sequence”) and a barcode generated for the purposes of building the BOLD reference library (“reference sequence”). A query sequence is generated in a laboratory setting and used as a search string in the BOLD Identification System to obtain a species-level match, wherever possible. Although a query sequence must include a minimum number of nucleotides from a designated barcode region [14] in order for a search to be performed via the BOLD Identification System, it is not subject to any specific data standards or scientific requirements. On the other hand, a reference sequence forms the molecular genetic component of a reference DNA barcode record in BOLD that includes other forms of detailed information associated with the source specimen and the collection event [14]. Because a reference barcode record constitutes the core data element in BOLD that enables accurate species identifications to be performed by end-users, the data contained in each record must meet a variety of formal data standards before publication in INSDC (Figure 4).

The submission console of BOLD-SDP was specifically designed for project leaders and members of the scientific community to review student compliance with these standards before their data is transferred to the BOLD researcher workbench and simultaneously published in INSDC (Figure 4). To ensure a greater level of student success in publishing their records, educators are therefore encouraged to familiarize themselves with current standards for specimen and sequence data before embarking upon a DNA barcoding project with their students (Figure 4) [14]. Educators are also encouraged to establish partnerships with taxonomists and other experts to verify species names associated with DNA barcode sequences and to identify a suitable biorepository to curate specimens (voucher specimens) from which barcode sequences were obtained. Apart from preserving the fidelity of BOLD as a global species identification tool, such partnerships will provide a source of important technical guidance and will expose students to a valuable aspect of biodiversity science, namely, the curation of specimens and the management of collections and collection data. In recognition of the obvious challenges that educators may face in forging these partnerships, the authors are currently establishing cyberinfrastructure to support the formation of a community of scientists and educators who share an interest in broadening the involvement of students in building the BOLD reference library as citizen scientists. A complete description of this effort will appear elsewhere.

## Implementation

The inherently interdisciplinary scope of the project's supporting curriculum makes it suitable for implementation in a variety of standard, honors-level, Advanced Placement, and International Baccalaureate life science courses in biology, chemistry, environmental science, marine biology, and biotechnology. In fact, science teachers participating in the program have successfully implemented the curriculum in each of these subject areas. The curriculum is also amenable to several distinct implementation strategies. For instance, some teachers compress a standard course curriculum to implement the project as a capstone experience at the end of a semester. Others place the barcoding project at the center of a new elective course that they develop in consultation with our scientific team, school administrators, and teacher colleagues. Another group of teachers utilizes the conceptual and technical foundation of the project curriculum to guide a substantive and creative restructuring of standard course curricula. Since a primary goal of the project is to facilitate a potentially transformative shift in in-school teaching practices that favors a more interdisciplinary and integrational approach to life science instruction, we regard the latter two strategies as particularly encouraging project outcomes.

## Educational Outcomes and Student Data Contributions

Formal evaluation surveys have been designed and administered to examine the program's direct and indirect impacts on the knowledge, attitudes, and behaviors of teacher and student participants (Text S1). In the current project year, teachers demonstrated statistically significant gains on each of six measures aimed at assessing direct impacts on general scientific knowledge, technical and laboratory knowledge, technical proficiency, teaching efficacy, and teaching practices. Students also showed statistically significant increases on measures aimed at evaluating direct impacts on science process knowledge and understanding of the scientific inquiry process. Results from paired sample *t*-tests further revealed that teachers and students achieved statistically significant learning gains in comprehensive measures of project-related content knowledge. A more complete description of evaluation results for the current project year, including sample survey items, indirect qualitative data obtained from students of teacher participants, and a list of project attributes that teachers regard as especially important for promoting student investment in the project, appears in Text S1.

In addition to its potential for promoting transformative positive learning outcomes, the engagement of students in creating reference DNA barcodes produces a body of new scientific information and genomic resources for the benefit of the broader scientific community. These scientific outcomes distinguish the project from alternative educational strategies that take as their primary scientific learning objective the identification of unknown specimens using the reference barcode records already contained in BOLD (an objective that, in many cases, will be complicated by incomplete taxonomic coverage of various groups within the BOLD repository).

**Figure 5:**
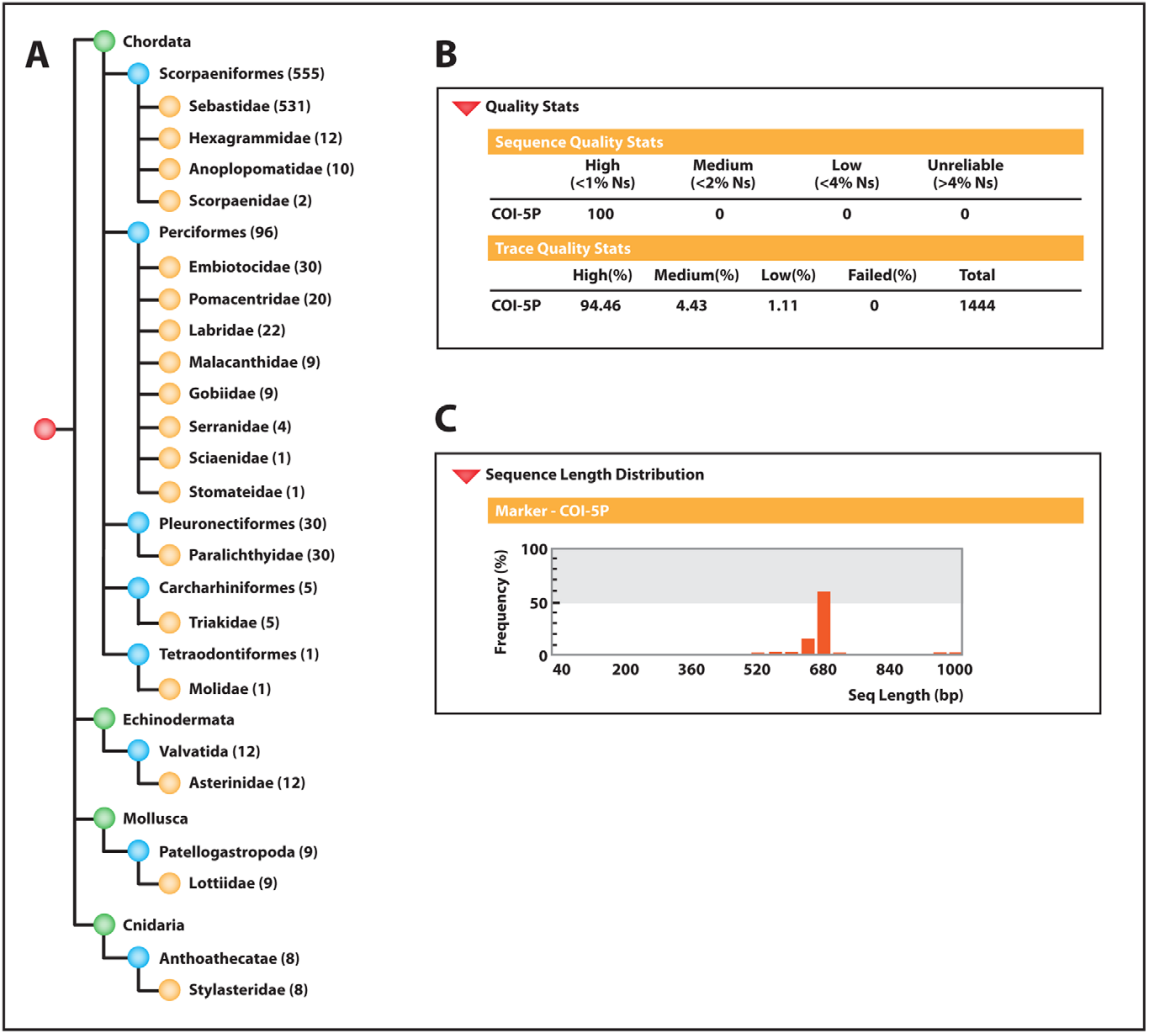
Reference DNA barcode records generated by project participants. (A) As of October 2012, students and teachers have generated and submitted complete reference DNA barcode records from 716 unique individuals representing four animal phyla, eight orders, 18 families, 26 genera (not shown), and 53 species (not shown). 716 records are currently published in GenBank (see Text S2 for the accession numbers corresponding to each published record). GenBank accession numbers, specimen and collection data, nucleotide sequences, trace files, and primer details are also available within the Barcoding Life's Matrix project folder, which is accessible through the BOLD Public Data Portal (http://www.boldsystems.org/index.php/Public_BINSearch?searchtype=records; use search term “BLM”). (B) Quality statistics for edited and unedited CO1 nucleotide sequence data. CO1 sequences edited by project participants from raw trace files contain no ambiguous base calls (Ns), stop codons, contaminating sequences, or insertions or deletions. Each amplicon was sequenced bidirectionally to yield at least one forward and one reverse trace file for each barcode record. Of the 1,444 trace files generated, 94.46% are categorized as high quality (mean Phred quality score >40 [28]), 4.43% are categorized as medium quality (mean Phred quality score = 30–40), and 1.11% are categorized as low quality (mean Phred quality score <30). (C) Nucleotide length distribution of CO1 sequences generated for each specimen. All 716 sequences generated by project participants exceeded the minimum barcode length of 500 nucleotides, with a minimum sequence length of 502 nucleotides, a maximum sequence length of 1,152 nucleotides, and a mean sequence length of 720 nucleotides.

Since its inception, the project has engaged over 1,000 high school students representing 60 California cities and seven states in generating and submitting 716 professional quality reference DNA barcode records to BOLD and INSDC (Figure 5). Through their efforts, students have created a valuable and comprehensive reference DNA barcode library for a targeted list of economically and commercially important marine taxa found in California's Channel Islands National Park and Marine Sanctuary. Furthermore, by depositing tissue samples from source voucher specimens in the Ocean Genome Resource of Ocean Genome Legacy, a marine research institute and genome bank in Ipswich, Massachusetts, students have also assembled a publicly accessible collection of marine genome resources for noncommercial research. To our knowledge, this student-led project represents the first systematic DNA-based inventory of species diversity in any US national park.

A series of recent studies demonstrates the value of applied DNA barcoding for examining important aspects of marine biodiversity, conservation, and ecology [23]–[27]. By creating a well-parameterized reference library of marine fish and invertebrate species, students participating in the Barcoding Life's Matrix program have assembled the key scientific resources needed for their successors to pursue advanced research projects aimed at understanding important aspects of kelp forest ecology. In conjunction with our scientific collaborators, efforts are now underway to engage students in a series of novel and authentic investigational studies that make use of this barcode dataset to explore larval settlement and recruitment dynamics around the Channel Islands Marine Protected Areas, cryptic speciation in marine fishes, kelp forest trophic interactions, and fish host–parasite interactions.

## Concluding Remarks

The Barcoding Life's Matrix project represents a striking new example of how discovery-based science can be effectively translated into secondary educational settings to address science education reform agendas, overcome extremely difficult challenges associated with molecular life science teaching and learning, and engage large numbers of students in the creation of a valuable public and scientific resource (an aspect of the project that students regard as particularly exciting and transformative). Importantly, the program also highlights how a discovery-based mode of instruction can provide a structured inroad to student-led molecular life science investigations that circumvents potentially insurmountable practical and pedagogical barriers to purely open-ended forms of research-based inquiry in high school settings. Because the project's interdisciplinary core curriculum is inherently flexible and adaptable to different implementation strategies in a variety of life science courses, it lends itself to broad-scale replication in both secondary and post-secondary educational settings.

## Supporting Information

Text S1 Evaluation data obtained for year 1 of the Barcoding Life's Matrix project.
(PDF)

Text S2 GenBank accession numbers for reference DNA barcode records published by Barcoding Life's Matrix project participants.
(PDF)

## Abbreviations

BOLD: Barcode of Life Data Systems
BOLD-SDP: Barcode of Life Data Systems Student Data Portal
iBOL: International Barcode of Life
INSDC: International Nucleotide Sequence Data Collaboration
