# Supplementary material for: Barcoding Life's Matrix: Translating Biodiversity Genomics into High School Settings to Enhance Life Science Education
Source: PLoS Biol. 2013 Jan 29;11(1):e1001471. doi: 10.1371/journal.pbio.1001471 (PMC3558426; doi:10.1371/journal.pbio.1001471)
Supplement: Text S1 — Evaluation data obtained for year 1 of the Barcoding Life's Matrix project. (PDF) [file pbio.1001471.s001.pdf]

CONTENT KNOWLEDGE (TEACHER AND STUDENT PARTICIPANTS)

Sample Content Questions [dichotomously scored items (correct: 1 incorrect: 0); score for each content area calculated using mean of items answered correctly]

Content Area 1

1. The DNA barcoding pipeline involves the following core molecular biology technique(s):
2. Why was a mitochondrial gene chosen for DNA barcoding of animal species? (Mark all that apply)

Content Area 2

1. What distinguishes the nucleotide subunits in DNA and RNA? (Mark all that apply)
2. Nucleotide differences in the barcode region of different species are most likely to occur at which codon position?

Content Area 3

1. Silica spin column purification of DNA represents which type of separation method? (Mark all that apply)
2. The purpose of the elution step in spin column DNA purification is to: (Mark all that apply)

Content Area 4

1. In gel electrophoresis, increasing the concentration of agarose will: (Mark all that apply)

2. The rate at which DNA fragments migrate through an agarose gel is determined by: (Mark all that apply)

Content Area 5

1. A transilluminator is used to: (Mark all that apply)

Content Area 6

1. Helicase is an enzyme that is used to: (Mark all that apply)

2. The targeted amplification of a specific DNA fragment by PCR is primarily a function of: (Mark all that apply)

Content Area 7

1. During spin column purification of a PCR product, the DNA may be eluted from the column using: (Mark all that apply)

2. Which of the following PCR reaction components are removed during spin column purification of PCR products? (Mark all that apply)

Content Area 8

1. An automated sequencing reaction contains which of the following components: (Mark all that apply)

2. The results of a sequencing reaction may be displayed in which of the following formats:

Content Area 9 (administered to students only)

1. The use of antibiotics during the subcloning process is to:

2. With respect to Taq polymerase, terminal transferase activity refers to:

Results (professional development workshop teacher participants)

Content Area (# items)	Pre		Post		Post-Pre		Paired-Sample t-test		
	mean	sd	mean	sd	mean incr.	sd	*t*	*df*	*p*
Content Area 1 (22)	.66	.11	.82	.12	.16	.11	6.21***	16	.000
Content Area 2 (23)	.67	.12	.78	.14	.11	.13	3.33**	16	.004
Content Area 3 (18)	.78	.14	.93	.07	.15	.13	5.00***	16	.000
Content Area 4 (23)	.77	.09	.84	.07	.07	.11	2.66*	16	.017
Content Area 5 (15)	.77	.10	.86	.07	.09	.10	3.55**	16	.003
Content Area 6 (22)	.80	.15	.87	.08	.07	.12	2.61*	16	.019
Content Area 7 (13)	.62	.14	.80	.11	.18	.15	5.01***	16	.000
Content Area 8 (27)	.60	.15	.65	.12	.05	.16	1.29	16	.217

*p<.05 **p<.01 ***p<.001

Supplementary Text S1

Results (residential research institute student participants)

Content Area (# items)	Pre		Post		Post-Pre		Paired-Sample t-test		
	mean	sd	mean	sd	mean incr.	sd	*t*	*df*	*p*
Content Area 1 (22)	.70	.11	.77	.06	.07	.11	2.35*	13	.035
Content Area 2 (23)	.63	.10	.71	.11	.08	.13	2.41*	13	.032
Content Area 3 (18)	.63	.12	.80	.14	.17	.16	4.01**	13	.001
Content Area 4 (23)	.54	.12	.74	.13	.21	.18	4.34**	13	.001
Content Area 5 (15)	.63	.14	.83	.10	.21	.17	4.47**	13	.001
Content Area 6 (22)	.69	.09	.81	.13	.12	.14	3.23**	13	.007
Content Area 7 (13)	.48	.11	.68	.15	.20	.19	4.06**	13	.001
Content Area 8 (27)	.51	.08	.58	.09	.07	.11	2.31*	13	.038
Content Area 9 (06)	.41	.16	.67	.21	.26	.14	6.90***	13	.000

*p<.05 **p<.01 ***p<.001

ATTITUDES AND PERCEPTIONS (TEACHER PARTICIPANTS)

Sample Questions [all 6-pt Likert-style scales ranging from strongly disagree (1) to strongly agree (6)]

Scientific Knowledge

1. I have sufficient knowledge to provide my students with a strong foundation in contemporary life science concepts.

2. I have sufficient knowledge to teach my students how scientific research is conducted in real-world settings.

General Technical Knowledge

1. I have a solid knowledge of laboratory techniques fundamental to molecular life science research.

2. My knowledge of life science research tools, protocols and research methodologies is current.

Technical Proficiency

1. How would you describe your current level of proficiency in:

    a. extracting genomic DNA
    b. performing agarose gel electrophoresis
    c. performing polymerase chain reaction
    d. using bioinformatics tool to analyze and edit nucleotide sequence data

Laboratory Knowledge

How would you describe your current level of level of understanding of:

a. agarose gel electrophoresis
b. polymerase chain reaction
c. nucleic acid purification
d. automated DNA sequencing

Teaching Efficacy

1. I can answer most scientific questions that my students ask.

2. I can prepare my students for a successful career in a science-related field.

Teaching Practices

1. I explain molecular biology procedures and protocols by the scientific principles upon which they are based.

2. I design investigations in which students explore scientific questions with modern tools of inquiry.

Results

Content Area (# items)	Pre		Post		Post-Pre		Paired-Sample t-test		
	mean	sd	mean	sd	mean incr.	sd	*t*	*df*	*p*
Scientific Knowledge (9)	4.78	0.68	5.10	0.55	0.31	0.54	2.37*	16	0.030
Technical Knowledge (5)	4.13	0.84	5.14	0.39	1.01	0.80	5.20***	16	0.000
Technical Proficiency (5)	3.69	1.19	5.41	0.49	1.72	0.91	7.80***	16	0.000
Lab Knowledge (4)	4.09	1.21	5.53	0.40	1.44	1.09	5.44***	16	0.000
Teaching Efficacy (18)	4.54	0.56	4.94	0.51	0.40	0.47	3.55**	16	0.003
Teaching Practices (10)	4.41	0.62	5.19	0.52	0.78	0.61	5.31***	16	0.000
*$p<.05$ **$p<.01$ ***$p<.001$									

ATTITUDES AND PERCEPTIONS (STUDENT PARTICIPANTS)

Sample Questions [all 6-pt Likert-style scales ranging from strongly disagree (1) to strongly agree (6)]

Knowledge

1. I can explain how the scientific discovery process unfolds in real-world research settings.
2. I am able to integrate knowledge from different scientific fields.

Efficacy

1. I am confident in my ability to understand complex scientific ideas and processes.
2. I do not know a lot about life science research tools, protocols, and research methodologies.

Scientific Inquiry

1. Using the scientific method helps me to reason through scientific problems.
2. Science informs my life choices, such as what to eat and which car to buy.

Results

Content Area (# items)	Pre		Post		Post-Pre		Paired-Sample t-test		
	mean	sd	mean	sd	mean incr.	sd	*t*	*df*	*p*
Knowledge (15)	4.36	0.47	4.97	0.32	0.61	0.35	6.44***	13	0.000
Efficacy (5)	4.60	0.52	4.54	0.38	0.06	0.25	0.84	13	0.414
Scientific Inquiry (26)	5.00	0.36	5.36	0.38	0.36	0.13	10.33***	13	0.000
*$p<.05$ **$p<.01$ ***$p<.001$									

QUALITATIVE FEEDBACK FROM STUDENT PARTICIPANTS

We present qualitative data in the form of informal feedback from high school students who participated in the project under the supervision of their teachers. This data represents a compilation of verbatim responses to an open-ended question that asked students to comment on their experience in the project and how it affected them, their career goals, and their perceptions of science. A subset of student responses was selected and categorized based on congruency with the project attributes and career constructs that teachers regarded as especially important for promoting student investment in, and excitement over, the project. A more complete list of student responses can be found on the Student Reflections page of the project website, which resides on the following URL: http://studentdnabarcoding.org/featured-participants/perspectives/student-reflections.html

Supplementary Text S1

1. Interdisciplinary Scope of Project Curriculum

a. “…probably the thing that affected me the most and that has changed my perspective on science was the fact that not only did we learn new lab techniques, but we learned and saw first hand how science brings ideas together and how scientific experiments are carried out.”

b. “Through the project, I gained an enormous appreciation for how different scientific fields are interconnected. While I knew that science fields are diverse and target many subjects, I never saw how these fields connect to one another.”

c. “… this program allowed me to observe how different aspects of nature, science and technology are intertwined in the scientific process.”

d. “I gained a lot of knowledge about the interconnectivity of field work, lab work, and technology.”

2. Relevance of Scientific Topics and Methods Taught in School to Real-World Problems

a. “This project has shown me how everything we're learning can be applied to real world problems, including applications in medicine.”

b. “[The program] showed me that some of the information I'm learning in school is actually useful in the real world.”

c. “It really opened my eyes up to what I can study in science for college and how I can use that knowledge to accomplish a wide variety of things that can be helpful in the future to society and the environment.”

d. “ one of the greatest things I learned is that learning isn't simply memorization but thinking critically and creatively and trying to apply what you know to real-world situations.

3. Authenticity of Project-Related Activities

a. "Participating in this lab series showed me some of the skills and techniques that a biodiversity genomics scientist might apply in their actual job; it was awesome to be able to conduct experiments that adult professionals also perform."

b. "This was the first time that I have felt that something I accomplished in school would impact the world of science."

c. "This experience has shown me what real science is like."

d. "I was doing real science, and I actually had a deep understanding of the science concepts involved and the global benefit of my research."

4. Contributions to the Scientific Community

a. " [the program provided] a one of a kind opportunity to contribute to a cause that directly benefits the world we live in."

b. "… our results have not only been published for the entire world to see, but to use in future experiments. I feel like I have accomplished something great."

c. "Being part of this global community of scientists trying to preserve the biodiversity of the planet is a rewarding feeling that cannot be matched. It is great to know the importance of biodiversity in the world and that I have taken a small part in helping to maintain it."

d. “It is a fantastic opportunity to do hands-on scientific research that actually contributes something to the scientific world …   You actually get to participate in the scientific process, and are able to collaborate in an international effort as an equal, not as an understudy”

5. Motivating a changed perspective on science

a. “Scientific research was not really in my “to do list”, but it should have been.”

b. “It was such a wonderful experience being a part of a larger scientific community. I now look at science in a completely new way.”

c. “CMB has definitely changed my perspective of science. It's not all about studying from textbooks, real learning is achieved by actually applying the concepts to real scientific research.”

d. “Not only did the barcoding project help stimulate my interest in biology; it also sparked a possible path into a scientific-related career.”

e. “Despite being interested in science, I have always imagined scientists to be individuals I would never be able to relate to. I was wrong.”
