# Supplementary material for: Barcoding Life's Matrix: Translating Biodiversity Genomics into High School Settings to Enhance Life Science Education
Source: PLoS Biol. 2013 Jan 29;11(1):e1001471. doi: 10.1371/journal.pbio.1001471 (PMC3558426; doi:10.1371/journal.pbio.1001471)
Supplement: Text S2 — GenBank accession numbers for reference DNA barcode records published by Barcoding Life's Matrix project participants. (PDF) [file pbio.1001471.s002.pdf]

Supplementary Text S2

| Species | Sample ID | GenBank Accession Number |
| --- | --- | --- |
| Amphistichus argenteus | NH-72 | JX487273 |
| Amphistichus argenteus | NH-73 | JX487275 |
| Amphistichus argenteus | NH-71 | JX487276 |
| Amphistichus argenteus | NH-74 | JX487274 |
| Anoplopoma fimbria | ANFI-08 | JX110779 |
| Anoplopoma fimbria | ANFI-05 | JX110777 |
| Anoplopoma fimbria | ANFI-07 | JX110793 |
| Anoplopoma fimbria | ANFI-09 | JX110780 |
| Anoplopoma fimbria | ANFI-06 | JX110778 |
| Anoplopoma fimbria | ANFI-04 | JX110776 |
| Anoplopoma fimbria | ANFI-10 | JX110781 |
| Anoplopoma fimbria | ANFI-02 | JX110791 |
| Anoplopoma fimbria | ANFI-03 | JX110792 |
| Anoplopoma fimbria | ANFI-01 | JX110790 |
| Caulolatilus princeps | VH-14 | JQ672529 |
| Caulolatilus princeps | VH-2 | JQ672517 |
| Caulolatilus princeps | VH-1 | JQ672516 |
| Caulolatilus princeps | VH-15 | JQ672530 |
| Caulolatilus princeps | VH-5 | JQ672520 |
| Caulolatilus princeps | VH-6 | JQ672521 |
| Caulolatilus princeps | VH-10 | JQ672525 |
| Caulolatilus princeps | VH-3 | JQ672518 |
| Caulolatilus princeps | VH-4 | JQ672519 |
| Chromis punctipinnis | CHPU-07 | JQ934972 |
| Chromis punctipinnis | VH-35 | JQ694039 |
| Chromis punctipinnis | VH-34 | JQ694038 |
| Chromis punctipinnis | CHPU-08 | JQ934976 |
| Chromis punctipinnis | VH-37 | JQ694041 |
| Chromis punctipinnis | VH-38 | PENDING |
| Chromis punctipinnis | CHPU-09 | JQ934975 |
| Chromis punctipinnis | CHPU-11 | JQ934966 |
| Chromis punctipinnis | CHPU-02 | JQ934973 |
| Chromis punctipinnis | CHPU-05 | JQ934974 |
| Chromis punctipinnis | CHPU-06 | JQ934971 |
| Citharichthys sordidus | VH-7 | JQ672522 |

| | | |
|---|---|---|
| Citharichthys sordidus | VH-17 | JQ672532 |
| Citharichthys sordidus | VH-23 | JQ694029 |
| Citharichthys sordidus | NH-44 | JX438502 |
| Citharichthys sordidus | NH-51 | JX438513 |
| Citharichthys sordidus | NH-45 | JX438507 |
| Citharichthys sordidus | NH-59 | JX438521 |
| Citharichthys sordidus | NH-46 | JX438503 |
| Citharichthys sordidus | NH-52 | JX438511 |
| Citharichthys sordidus | NH-60 | JX438525 |
| Citharichthys sordidus | NH-47 | JX438508 |
| Citharichthys sordidus | NH-12 | JX295815 |
| Citharichthys sordidus | NH-13 | JX295822 |
| Citharichthys sordidus | NH-61 | JX438522 |
| Citharichthys sordidus | NH-48 | JX438512 |
| Citharichthys sordidus | NH-11 | JX295817 |
| Citharichthys sordidus | NH-50 | JX438509 |
| Citharichthys sordidus | NH-55 | JX438519 |
| Citharichthys sordidus | NH-56 | JX438515 |
| Citharichthys sordidus | NH-42 | JX438505 |
| Citharichthys sordidus | NH-43 | JX438506 |
| Citharichthys sordidus | NH-22 | JX295825 |
| Citharichthys sordidus | VH-13 | JQ672528 |
| Citharichthys sordidus | VH-19 | JQ672534 |
| Citharichthys sordidus | VH-18 | JQ672533 |
| Citharichthys sordidus | VH-12 | JQ672527 |
| Citharichthys sordidus | VH-16 | JQ672531 |
| Citharichthys sordidus | VH-9 | JQ672524 |
| Citharichthys sordidus | VH-8 | JQ672523 |
| Citharichthys sordidus | VH-11 | JQ672526 |
| Embiotoca jacksoni | VH-22 | JQ694028 |
| Embiotoca jacksoni | VH-21 | JQ694027 |
| Embiotoca jacksoni | EMJA-03 | JN582132 |
| Embiotoca jacksoni | EMJA-01 | JN582121 |
| Embiotoca jacksoni | EMJA-04 | JN582131 |
| Embiotoca jacksoni | EMJA-02 | JN582120 |
| Embiotoca lateralis | EMLA-07 | JN582111 |
| Embiotoca lateralis | EMLA-09 | JN582093 |

| | | |
|---|---|---|
| Embiotoca lateralis | EMLA-10 | JN582094 |
| Embiotoca lateralis | EMLA-05 | JN582102 |
| Embiotoca lateralis | EMLA-06 | JN582103 |
| Embiotoca lateralis | EMLA-08 | JN582112 |
| Embiotoca lateralis | EMLA-01 | JN582084 |
| Embiotoca lateralis | EMLA-02 | JN582085 |
| Embiotoca lateralis | EMLA-04 | JN582086 |
| Genyonemus lineatus | VN-1 | JQ934981 |
| Halichoeres semicinctus | HASE-07 | JX124728 |
| Halichoeres semicinctus | HASE-06 | JX124730 |
| Halichoeres semicinctus | HASE-05 | JX124729 |
| Halichoeres semicinctus | HASE-08 | JX124731 |
| Hypsypops rubicundus | HYRU-01 | JN582165 |
| Hypsypops rubicundus | HYRU-03 | JN582163 |
| Hypsypops rubicundus | HYRU-10 | JX487230 |
| Hypsypops rubicundus | HYRU-02 | JN582164 |
| Hypsypops rubicundus | HYRU-07 | JN582167 |
| Hypsypops rubicundus | HYRU-09 | JX487229 |
| Hypsypops rubicundus | HYRU-04 | JN582170 |
| Hypsypops rubicundus | HYRU-05 | JN582169 |
| Hypsypops rubicundus | HYRU-06 | JN582168 |
| Hypsypops rubicundus | HYRU-08 | JN600313 |
| Lottia gigantea | OGL-E01620 | JF433977 |
| Lottia gigantea | KFM-217 | JF433981 |
| Lottia gigantea | OGL-E01615 | JF433978 |
| Lottia gigantea | KFM-216 | JF433982 |
| Lottia gigantea | OGL-E01605 | JF433980 |
| Lottia gigantea | OGL-E01613 | JF433979 |
| Lottia gigantea | OGL-E01622 | JF433976 |
| Lottia gigantea | OGL-E01590 | JF433975 |
| Lottia gigantea | OGL-E01594 | JF433974 |
| Mola mola | NH-54 | JX438518 |
| Mustelus henlei | GE-7 | JX495633 |
| Mustelus henlei | GE-6 | JX495631 |
| Mustelus henlei | GE-2 | JX495621 |
| Mustelus henlei | GE-1a | JX495616 |
| Mustelus henlei | GE-4 | JX495628 |

| | | |
|---|---|---|
| Ophiodon elongatus | NH-30 | JX295831 |
| Oxyjulis californica | OXCA-04 | JN582148 |
| Oxyjulis californica | OXCA-05 | JN582147 |
| Oxyjulis californica | OXCA-07 | JN582145 |
| Oxyjulis californica | OXCA-01 | JN582151 |
| Oxyjulis californica | OXCA-02 | JN582150 |
| Oxyjulis californica | OXCA-10 | JN582142 |
| Oxyjulis californica | OXCA-08 | JN582144 |
| Oxyjulis californica | OXCA-06 | JN582146 |
| Oxyjulis californica | OXCA-09 | JN582143 |
| Oxyjulis californica | OXCA-03 | JN582149 |
| Oxylebius pictus | OXPI-11 | JN582110 |
| Oxylebius pictus | OXPI-01 | JN582095 |
| Oxylebius pictus | OXPI-02 | JN582096 |
| Oxylebius pictus | OXPI-04 | JN582098 |
| Oxylebius pictus | OXPI-05 | JN582104 |
| Oxylebius pictus | OXPI-06 | JN582105 |
| Oxylebius pictus | OXPI-07 | JN582106 |
| Oxylebius pictus | OXPI-08 | JN582107 |
| Oxylebius pictus | OXPI-09 | JN582108 |
| Oxylebius pictus | OXPI-10 | JN582109 |
| Oxylebius pictus | OXPI-03 | JN582097 |
| Paralabrax clathratus | PACL-07 | JN582134 |
| Paralabrax clathratus | PACL-09 | JN600314 |
| Paralabrax clathratus | PACL-06 | JN600315 |
| Paralabrax clathratus | PACL-08 | JN582133 |
| Patiria miniata | KFM-426 | HQ231385 |
| Patiria miniata | KFM-432 | HQ231375 |
| Patiria miniata | KFM-420 | HQ231383 |
| Patiria miniata | KFM-419 | HQ231382 |
| Patiria miniata | KFM-415 | HQ231386 |
| Patiria miniata | KFM-423 | HQ231388 |
| Patiria miniata | KFM-416 | HQ231387 |
| Patiria miniata | KFM-417 | HQ231374 |
| Patiria miniata | KFM-431 | HQ231373 |
| Patiria miniata | KFM-429 | HQ231381 |
| Patiria miniata | KFM-425 | HQ231384 |

| | | |
|---|---|---|
| Patiria miniata | KFM-422 | HQ231380 |
| Peprilus simillimus | NH-6 | JX295807 |
| Phanerodon atripes | LO-37 | JX008537 |
| Rhacochilus vacca | DAVA-05 | JN582126 |
| Rhacochilus vacca | DAVA-04 | JN582127 |
| Rhacochilus vacca | DAVA-03 | JN582128 |
| Rhacochilus vacca | DAVA-01 | JN582130 |
| Rhacochilus vacca | DAVA-06 | JN582125 |
| Rhacochilus vacca | DAVA-02 | JN582129 |
| Rhacochilus vacca | DAVA-10 | JN582166 |
| Rhacochilus vacca | DAVA-09 | JN582122 |
| Rhacochilus vacca | DAVA-08 | JN582123 |
| Rhacochilus vacca | DAVA-07 | JN582124 |
| Rhinogobiops nicholsii | CONI-07 | JN582118 |
| Rhinogobiops nicholsii | CONI-01 | JN582113 |
| Rhinogobiops nicholsii | CONI-06 | JN582117 |
| Rhinogobiops nicholsii | CONI-10 | JN582087 |
| Rhinogobiops nicholsii | CONI-08 | JN582119 |
| Rhinogobiops nicholsii | CONI-09 | JN582135 |
| Rhinogobiops nicholsii | CONI-02 | JN582114 |
| Rhinogobiops nicholsii | CONI-04 | JN582116 |
| Rhinogobiops nicholsii | CONI-03 | JN582115 |
| Scorpaena guttata | NH-31 | JX295842 |
| Scorpaena guttata | NH-15 | JX295819 |
| Sebastes atrovirens | SE-33 | JN582160 |
| Sebastes atrovirens | SE-32 | JN582157 |
| Sebastes atrovirens | SZ-1 | HM107288 |
| Sebastes atrovirens | AJ-3 | GQ355759 |
| Sebastes atrovirens | SE-38 | JN571508 |
| Sebastes atrovirens | SE-35 | JN582161 |
| Sebastes atrovirens | VH-20 | JQ672535 |
| Sebastes atrovirens | NH-37 | JX438501 |
| Sebastes atrovirens | RE-32 | JQ952611 |
| Sebastes atrovirens | NH-1 | JX295812 |
| Sebastes atrovirens | RE-2 | JQ672537 |
| Sebastes atrovirens | RE-5 | JQ672539 |
| Sebastes atrovirens | VH-31 | JQ694035 |

| | | |
|---|---|---|
| Sebastes atrovirens | SE-31 | JN582158 |
| Sebastes atrovirens | SE-34 | JN582159 |
| Sebastes atrovirens | SEAT-10 | JN600316 |
| Sebastes auriculatus | SE-11 | JN571501 |
| Sebastes auriculatus | GMS-1 | HM107296 |
| Sebastes auriculatus | BHP-2 | HM107303 |
| Sebastes auriculatus | GMS-2 | HM107302 |
| Sebastes auriculatus | SE-29 | JN571517 |
| Sebastes auriculatus | SE-26 | JN571509 |
| Sebastes auriculatus | SE-10 | JN571500 |
| Sebastes auriculatus | SE-12 | JN571499 |
| Sebastes auriculatus | SE-20 | JN582155 |
| Sebastes auriculatus | SE-28 | JN571516 |
| Sebastes auriculatus | SE-27 | JN571515 |
| Sebastes auriculatus | SE-21 | JN571513 |
| Sebastes auriculatus | SE-04 | JN582154 |
| Sebastes auriculatus | SE-06 | JN582153 |
| Sebastes auriculatus | VN-13 | JQ934993 |
| Sebastes auriculatus | VN-19 | JQ934999 |
| Sebastes auriculatus | SE-09 | JN571498 |
| Sebastes auriculatus | SE-08 | JN571497 |
| Sebastes auriculatus | SE-07 | JN571496 |
| Sebastes auriculatus | SE-05 | JN571495 |
| Sebastes auriculatus | SE-03 | JN571494 |
| Sebastes auriculatus | SE-22 | JN571512 |
| Sebastes auriculatus | SE-23 | JN571511 |
| Sebastes auriculatus | SE-02 | JN571493 |
| Sebastes auriculatus | SE-30 | JN571518 |
| Sebastes auriculatus | SE-19 | JN582156 |
| Sebastes auriculatus | SE-24 | JN571510 |
| Sebastes auriculatus | SE-18 | JN571507 |
| Sebastes auriculatus | SE-17 | JN571506 |
| Sebastes auriculatus | SE-15 | JN571504 |
| Sebastes auriculatus | SE-14 | JN571503 |
| Sebastes auriculatus | SE-13 | JN571502 |
| Sebastes carnatus | LLM-1 | HM107301 |
| Sebastes carnatus | AM-2 | HM107292 |

| | | |
|---|---|---|
| Sebastes carnatus | RS-4 | GQ501097 |
| Sebastes carnatus | NH-3 | JX295808 |
| Sebastes carnatus | SE-36 | JN582162 |
| Sebastes carnatus | RS-1 | GQ501098 |
| Sebastes carnatus | NH-38 | JX438498 |
| Sebastes carnatus | NH-39 | JX438499 |
| Sebastes carnatus | BG-8 | GQ501100 |
| Sebastes carnatus | BG-9 | GQ497753 |
| Sebastes carnatus | LT-1 | HM107300 |
| Sebastes carnatus | NH-29 | JX295836 |
| Sebastes carnatus | RS-6 | GQ501096 |
| Sebastes caurinus | NH-21 | JX295826 |
| Sebastes caurinus | NH-66 | JX438528 |
| Sebastes caurinus | AA-1 | GQ254415 |
| Sebastes caurinus | NH-14 | JX295824 |
| Sebastes caurinus | NH-41 | JX438504 |
| Sebastes caurinus | BG-3 | GQ501104 |
| Sebastes caurinus | HA-5 | FJ501250 |
| Sebastes caurinus | NH-53 | JX438514 |
| Sebastes caurinus | NH-32 | JX295839 |
| Sebastes caurinus | AC-2 | HM107289 |
| Sebastes caurinus | NH-4 | JX295809 |
| Sebastes caurinus | NH-40 | JX438500 |
| Sebastes caurinus | FT-71 | JX110802 |
| Sebastes chlorostictus | FT-69 | JX487224 |
| Sebastes chlorostictus | FT-60 | JX487221 |
| Sebastes chlorostictus | HH-2 | HQ229647 |
| Sebastes chlorostictus | BLM-1 | HQ229645 |
| Sebastes chlorostictus | HH-1 | HQ229646 |
| Sebastes chlorostictus | BS-11 | GQ396756 |
| Sebastes chlorostictus | FKM-1 | HM107297 |
| Sebastes chlorostictus | BS-12 | GQ396757 |
| Sebastes chlorostictus | SE-50 | JN582073 |
| Sebastes chlorostictus | CMB-10 | HQ231393 |
| Sebastes chlorostictus | FT-26 | JX008504 |
| Sebastes chlorostictus | BS-19 | GQ396763 |
| Sebastes chlorostictus | BS-10 | GQ396755 |

| | | |
|---|---|---|
| Sebastes chlorostictus | SE-60 | JN582078 |
| Sebastes chlorostictus | AHL-2 | HQ229642 |
| Sebastes chlorostictus | CMB-2 | HQ231378 |
| Sebastes chlorostictus | SE-48 | JN582071 |
| Sebastes chlorostictus | SE-47 | JN582070 |
| Sebastes chlorostictus | NH-76 | JX444757 |
| Sebastes chlorostictus | NH-86 | JX444765 |
| Sebastes chlorostictus | NH-87 | JX444764 |
| Sebastes chlorostictus | NH-98 | JX444773 |
| Sebastes chlorostictus | NH-78 | JX444761 |
| Sebastes chlorostictus | NH-99 | JX444775 |
| Sebastes chlorostictus | NH-79 | JX444760 |
| Sebastes chlorostictus | NH-89 | JX444769 |
| Sebastes chlorostictus | NH-100 | JX444771 |
| Sebastes chlorostictus | NH-90 | JX444768 |
| Sebastes chlorostictus | NH-80 | JX444759 |
| Sebastes chlorostictus | NH-101 | JX444778 |
| Sebastes chlorostictus | NH-91 | JX444770 |
| Sebastes chlorostictus | NH-81 | JX444763 |
| Sebastes chlorostictus | NH-93 | JX444767 |
| Sebastes chlorostictus | NH-113 | JX444783 |
| Sebastes chlorostictus | NH-115 | JX444785 |
| Sebastes chlorostictus | NH-106 | JX444780 |
| Sebastes chlorostictus | NH-97 | JX495623 |
| Sebastes chlorostictus | NH-108 | JX495627 |
| Sebastes chlorostictus | NH-85 | JX495618 |
| Sebastes chlorostictus | NH-107 | JX495624 |
| Sebastes chlorostictus | NH-118 | JX495632 |
| Sebastes chlorostictus | FT-68 | JX110801 |
| Sebastes chlorostictus | FT-47 | JX110810 |
| Sebastes chlorostictus | FT-44 | JX110809 |
| Sebastes chlorostictus | FT-48 | JX110811 |
| Sebastes chrysomelas | CN-2 | FJ501251 |
| Sebastes chrysomelas | VH-33 | JQ694037 |
| Sebastes chrysomelas | VH-32 | JQ694036 |
| Sebastes chrysomelas | RL-4 | GQ355771 |
| Sebastes chrysomelas | SE-01 | JN571492 |

| | | |
|---|---|---|
| Sebastes constellatus | GT-2 | GQ355768 |
| Sebastes constellatus | AB-A-Cox1 | FJ158547 |
| Sebastes constellatus | PS-03 | GQ254302 |
| Sebastes constellatus | PS-01 | GQ254303 |
| Sebastes constellatus | LO-28 | JX008529 |
| Sebastes constellatus | LO-29 | JX487231 |
| Sebastes constellatus | LO-26 | JX008527 |
| Sebastes constellatus | LO-31 | JX008531 |
| Sebastes constellatus | LO-32 | JX008532 |
| Sebastes constellatus | LO-33 | JX008533 |
| Sebastes constellatus | LO-34 | JX008534 |
| Sebastes constellatus | LO-35 | JX008535 |
| Sebastes constellatus | BZ-3 | GQ355765 |
| Sebastes constellatus | BM-2 | GQ355762 |
| Sebastes constellatus | LO-30 | JX008530 |
| Sebastes constellatus | LO-36 | JX008536 |
| Sebastes constellatus | LO-27 | JX008528 |
| Sebastes crocotulus | FT-41 | JX487269 |
| Sebastes dallii | RE-37 | JQ952616 |
| Sebastes dallii | RE-33 | JQ952613 |
| Sebastes dallii | RE-27 | JQ952608 |
| Sebastes dallii | RE-30 | JQ952609 |
| Sebastes dallii | RE-26 | JQ952606 |
| Sebastes dallii | RE-21 | JQ952602 |
| Sebastes dallii | RE-1 | JQ672536 |
| Sebastes dallii | RE-3 | JQ672538 |
| Sebastes dallii | RE-9 | JQ672542 |
| Sebastes dallii | RE-10 | JQ672545 |
| Sebastes dallii | RE-4 | JQ672540 |
| Sebastes dallii | RE-17 | JQ672552 |
| Sebastes dallii | RE-18 | JQ672551 |
| Sebastes dallii | RE-19 | JQ672554 |
| Sebastes dallii | RE-6 | JQ672541 |
| Sebastes dallii | RE-20 | JQ672555 |
| Sebastes dallii | RE-7 | JQ672543 |
| Sebastes dallii | RE-8 | JQ672544 |
| Sebastes dallii | SE-41 | JN582067 |

| | | |
|---|---|---|
| Sebastes dallii | RE-36 | JX124732 |
| Sebastes dallii | RE-28 | JX124733 |
| Sebastes dallii | RE-25 | JQ952607 |
| Sebastes dallii | RE-24 | JQ952605 |
| Sebastes dallii | RE-23 | JQ952604 |
| Sebastes dallii | RE-22 | JQ952603 |
| Sebastes dallii | RE-31 | JQ952612 |
| Sebastes elongatus | BKO-1 | HM107304 |
| Sebastes elongatus | NH-111 | JX495630 |
| Sebastes elongatus | NH-116 | JX444784 |
| Sebastes elongatus | NH-110 | JX444779 |
| Sebastes ensifer | BS-7 | GQ396752 |
| Sebastes ensifer | CMB-16 | HQ231389 |
| Sebastes ensifer | NH-84 | JX444766 |
| Sebastes ensifer | NH-92 | JX495617 |
| Sebastes ensifer | CMB-14 | HQ231391 |
| Sebastes ensifer | CMB-20 | HQ229644 |
| Sebastes ensifer | PS-05 | GQ254304 |
| Sebastes ensifer | PS-02 | GQ254305 |
| Sebastes ensifer | BS-9 | GQ396758 |
| Sebastes goodei | FT-29 | JX008507 |
| Sebastes goodei | FT-27 | JX008505 |
| Sebastes goodei | FT-19 | JX487265 |
| Sebastes goodei | FT-31 | JX008509 |
| Sebastes goodei | FT-20 | JX487263 |
| Sebastes goodei | FT-10 | JX487261 |
| Sebastes goodei | FT-39 | JX008515 |
| Sebastes goodei | FT-9 | JX487257 |
| Sebastes goodei | FT-33 | JX008511 |
| Sebastes goodei | FT-40 | JX008516 |
| Sebastes goodei | NH-49 | JX438510 |
| Sebastes goodei | FT-15 | JX487260 |
| Sebastes goodei | FT-14 | JX008500 |
| Sebastes goodei | FT-17 | JX487262 |
| Sebastes goodei | FT-18 | JX008502 |
| Sebastes goodei | FT-4 | JX008517 |
| Sebastes goodei | FT-8 | JX008521 |

| | | |
|---|---|---|
| Sebastes goodei | FT-16 | JX008501 |
| Sebastes goodei | FT-6 | JX008519 |
| Sebastes goodei | FT-25 | JX487267 |
| Sebastes goodei | FT-7 | JX008520 |
| Sebastes goodei | FT-23 | JX487264 |
| Sebastes goodei | NH-112 | JX444776 |
| Sebastes goodei | NH-94 | JX444774 |
| Sebastes goodei | NH-95 | JX444772 |
| Sebastes goodei | NH-69 | JX438531 |
| Sebastes goodei | NH-117 | JX444781 |
| Sebastes goodei | BS-1 | GQ396748 |
| Sebastes goodei | NH-58 | JX438517 |
| Sebastes goodei | NH-103 | JX495625 |
| Sebastes goodei | CJ-3 | HM622589 |
| Sebastes goodei | SE-55 | JN582076 |
| Sebastes goodei | BS-2 | GQ396749 |
| Sebastes goodei | PS-04 | GQ254306 |
| Sebastes goodei | NH-82 | JX495619 |
| Sebastes goodei | FT-77 | JX110804 |
| Sebastes goodei | NH-109 | JX495629 |
| Sebastes goodei | NH-102 | JX495622 |
| Sebastes goodei | FT-30 | JX008508 |
| Sebastes goodei | FT-21 | JX008503 |
| Sebastes goodei | FT-37 | JX008513 |
| Sebastes goodei | FT-22 | JX008539 |
| Sebastes goodei | FT-34 | JX487268 |
| Sebastes hopkinsi | LO-40 | JX487233 |
| Sebastes hopkinsi | GHL-1 | HM107298 |
| Sebastes hopkinsi | RL-2 | GQ355773 |
| Sebastes hopkinsi | AR-2 | GQ260056 |
| Sebastes hopkinsi | AR-1 | GQ260057 |
| Sebastes hopkinsi | NH-114 | JX444782 |
| Sebastes hopkinsi | BZ-2 | GQ355766 |
| Sebastes hopkinsi | CMB-9 | HQ231394 |
| Sebastes hopkinsi | AB-D-Cox1 | FJ158548 |
| Sebastes hopkinsi | CMB-1 | HQ231379 |
| Sebastes hopkinsi | GM-4 | GQ254406 |

| | | |
|---|---|---|
| Sebastes hopkinsi | PS-08 | GQ254307 |
| Sebastes hopkinsi | IS-4 | GQ254402 |
| Sebastes hopkinsi | RL-3 | GQ355772 |
| Sebastes hopkinsi | BM-3 | GQ355763 |
| Sebastes hopkinsi | NH-65 | JX438530 |
| Sebastes hopkinsi | NH-67 | JX438527 |
| Sebastes melanostomus | SV-19 | JX487215 |
| Sebastes melanostomus | SV-15 | JX487212 |
| Sebastes melanostomus | SV-23 | JX487244 |
| Sebastes melanostomus | SV-24 | JX487248 |
| Sebastes melanostomus | SV-25 | JX487247 |
| Sebastes melanostomus | SV-26 | JX487246 |
| Sebastes melanostomus | SV-27 | JX487251 |
| Sebastes melanostomus | SV-22 | JX487243 |
| Sebastes melanostomus | SV-18 | JX487238 |
| Sebastes melanostomus | SV-21 | JX487241 |
| Sebastes melanostomus | SV-17 | JX487214 |
| Sebastes melanostomus | SV-16 | JX487213 |
| Sebastes melanostomus | SV-20 | JX487216 |
| Sebastes miniatus | RE-12 | JQ672547 |
| Sebastes miniatus | RE-13 | JQ672548 |
| Sebastes miniatus | SE-56 | JN582080 |
| Sebastes miniatus | SE-54 | JN582079 |
| Sebastes miniatus | CN-6 | FJ501252 |
| Sebastes miniatus | SE-53 | JN582077 |
| Sebastes miniatus | FT-52 | JX110814 |
| Sebastes miniatus | FT-55 | JX487217 |
| Sebastes miniatus | FT-12 | JX008499 |
| Sebastes miniatus | RE-39 | JX124734 |
| Sebastes miniatus | FT-43 | JX110808 |
| Sebastes miniatus | SE-25 | JN571514 |
| Sebastes miniatus | LO-38 | JX008538 |
| Sebastes miniatus | BS-5 | GQ396753 |
| Sebastes miniatus | FT-79 | JX110806 |
| Sebastes miniatus | GM-1 | GQ254407 |
| Sebastes miniatus | BZ-1 | GQ355767 |
| Sebastes miniatus | FT-36 | JX487266 |

| | | |
|---|---|---|
| Sebastes miniatus | RE-16 | JQ672553 |
| Sebastes miniatus | RE-38 | JQ952617 |
| Sebastes miniatus | FT-49 | JX487270 |
| Sebastes miniatus | RE-40 | JQ952618 |
| Sebastes miniatus | RE-35 | JQ952615 |
| Sebastes miniatus | SE-44 | JN582136 |
| Sebastes miniatus | RE-34 | JQ952614 |
| Sebastes miniatus | RE-15 | JQ672549 |
| Sebastes miniatus | RE-29 | JQ952610 |
| Sebastes miniatus | FT-63 | JX110788 |
| Sebastes miniatus | SE-16 | JN571505 |
| Sebastes miniatus | RE-14 | JQ672550 |
| Sebastes miniatus | IS-1 | GQ254403 |
| Sebastes miniatus | SE-52 | JN582075 |
| Sebastes miniatus | SE-43 | JN582069 |
| Sebastes miniatus | FT-51 | JX110813 |
| Sebastes miniatus | SE-42 | JN582068 |
| Sebastes miniatus | LO-39 | JX487234 |
| Sebastes mystinus | 1-BC | GQ254413 |
| Sebastes mystinus | SEMY-02 | JN582089 |
| Sebastes mystinus | 2-BC | GQ254412 |
| Sebastes mystinus | RA-2 | HM622582 |
| Sebastes mystinus | AR-3 | GQ260058 |
| Sebastes mystinus | AA-2 | GQ254417 |
| Sebastes mystinus | AA-3 | GQ254416 |
| Sebastes mystinus | SEMY-10 | JN582101 |
| Sebastes mystinus | VH-29 | JQ694042 |
| Sebastes mystinus | SEMY-01 | JN582088 |
| Sebastes mystinus | VH-26 | JQ694032 |
| Sebastes mystinus | SEMY-06 | JN582137 |
| Sebastes mystinus | SEMY-03 | JN582090 |
| Sebastes mystinus | SEMY-04 | JN582091 |
| Sebastes mystinus | SEMY-07 | JN582092 |
| Sebastes mystinus | SEMY-05 | JN582138 |
| Sebastes mystinus | VH-30 | JQ694034 |
| Sebastes mystinus | SEMY-08 | JN582099 |
| Sebastes mystinus | VH-28 | JQ694033 |

| | | |
|---|---|---|
| Sebastes mystinus | VH-25 | JQ694031 |
| Sebastes mystinus | SEMY-09 | JN582100 |
| Sebastes mystinus | GM-2 | GQ254408 |
| Sebastes ovalis | BM-1 | GQ355764 |
| Sebastes ovalis | BS-17 | GQ396764 |
| Sebastes ovalis | AR-4 | GQ260059 |
| Sebastes ovalis | LO-23 | JX008524 |
| Sebastes ovalis | LO-25 | JX008526 |
| Sebastes ovalis | LO-21 | JX008522 |
| Sebastes ovalis | LO-22 | JX008523 |
| Sebastes ovalis | LO-24 | JX008525 |
| Sebastes ovalis | BS-14 | GQ396760 |
| Sebastes ovalis | BS-13 | GQ396759 |
| Sebastes ovalis | BS-6 | GQ396754 |
| Sebastes ovalis | AA-4 | GQ254418 |
| Sebastes ovalis | LO-7 | JX050975 |
| Sebastes ovalis | LO-9 | JX050977 |
| Sebastes ovalis | LO-4 | JX050972 |
| Sebastes ovalis | LO-6 | JX050974 |
| Sebastes ovalis | LO-5 | JX050973 |
| Sebastes ovalis | LO-8 | JX050976 |
| Sebastes ovalis | CMB-6 | HQ231376 |
| Sebastes paucispinis | FT-72 | JX110803 |
| Sebastes paucispinis | FT-1 | JX487259 |
| Sebastes paucispinis | FT-42 | JX487272 |
| Sebastes paucispinis | FT-45 | JX487271 |
| Sebastes paucispinis | NH-104 | JX495626 |
| Sebastes paucispinis | NH-105 | JX444777 |
| Sebastes paucispinis | NH-77 | JX444758 |
| Sebastes paucispinis | FT-11 | JX008498 |
| Sebastes paucispinis | FT-5 | JX008518 |
| Sebastes paucispinis | FT-38 | JX008514 |
| Sebastes paucispinis | FT-28 | JX008506 |
| Sebastes paucispinis | FT-35 | JX008512 |
| Sebastes paucispinis | FT-32 | JX008510 |
| Sebastes paucispinis | LO-1 | JX050969 |
| Sebastes paucispinis | LO-3 | JX050971 |

| | | |
|---|---|---|
| Sebastes paucispinis | LO-2 | JX050970 |
| Sebastes paucispinis | FT-65 | JX110799 |
| Sebastes paucispinis | FT-80 | JX110807 |
| Sebastes paucispinis | FT-62 | JX110787 |
| Sebastes paucispinis | FT-78 | JX110805 |
| Sebastes paucispinis | FT-61 | JX110797 |
| Sebastes paucispinis | FT-50 | JX110812 |
| Sebastes paucispinis | FT-74 | JX487225 |
| Sebastes paucispinis | SE-46 | JN582139 |
| Sebastes paucispinis | GM-3 | GQ254409 |
| Sebastes paucispinis | IS-3 | GQ254404 |
| Sebastes paucispinis | IS-2 | GQ254405 |
| Sebastes paucispinis | SE-49 | JN582072 |
| Sebastes paucispinis | SE-58 | JN582083 |
| Sebastes paucispinis | SE-59 | JN582082 |
| Sebastes paucispinis | SE-57 | JN582081 |
| Sebastes paucispinis | GT-1 | GQ355769 |
| Sebastes paucispinis | SE-51 | JN582074 |
| Sebastes paucispinis | FT-75 | JX487228 |
| Sebastes paucispinis | FT-58 | JX487222 |
| Sebastes paucispinis | FT-57 | JX487219 |
| Sebastes paucispinis | NH-33 | JX295840 |
| Sebastes paucispinis | NH-34 | JX295838 |
| Sebastes paucispinis | NH-35 | JX295841 |
| Sebastes phillipsi | SV-28 | JX487252 |
| Sebastes phillipsi | SV-2 | JX487240 |
| Sebastes phillipsi | SV-4 | JX487237 |
| Sebastes phillipsi | SV-8 | JX487205 |
| Sebastes phillipsi | SV-6 | JX487203 |
| Sebastes phillipsi | SV-5 | JX487202 |
| Sebastes phillipsi | SV-3 | JX487201 |
| Sebastes phillipsi | SV-10 | JX487207 |
| Sebastes phillipsi | SV-9 | JX487206 |
| Sebastes phillipsi | SV-30 | JX487255 |
| Sebastes phillipsi | SV-29 | JX487249 |
| Sebastes phillipsi | SV-7 | JX487204 |
| Sebastes rastrelliger | VN-4 | JQ934984 |

| | | |
|---|---|---|
| Sebastes rastrelliger | VN-3 | JQ934983 |
| Sebastes rastrelliger | VN-2 | JQ934982 |
| Sebastes rastrelliger | VN-17 | JQ934997 |
| Sebastes rastrelliger | VN-18 | JQ934998 |
| Sebastes rastrelliger | VN-12 | JQ934992 |
| Sebastes rastrelliger | VN-11 | JQ934991 |
| Sebastes rastrelliger | VN-10 | JQ934990 |
| Sebastes rastrelliger | VN-9 | JQ934989 |
| Sebastes rastrelliger | VN-8 | JQ934988 |
| Sebastes rastrelliger | VN-7 | JQ934987 |
| Sebastes rastrelliger | VN-6 | JQ934986 |
| Sebastes rastrelliger | VN-20 | JX487227 |
| Sebastes rastrelliger | VN-16 | JQ934996 |
| Sebastes rastrelliger | VN-15 | JQ934995 |
| Sebastes rastrelliger | VN-14 | JQ934994 |
| Sebastes rastrelliger | VN-5 | JQ934985 |
| Sebastes rosaceus | NH-18 | JX295820 |
| Sebastes rosaceus | BG-10 | GQ497754 |
| Sebastes rosaceus | BG-1 | GQ501107 |
| Sebastes rosaceus | BG-4 | GQ501105 |
| Sebastes rosaceus | CB-1 | HM622587 |
| Sebastes rosaceus | PS-09 | GQ254308 |
| Sebastes rosaceus | PS-06 | GQ254309 |
| Sebastes rosaceus | AAE-1 | HQ229648 |
| Sebastes rosaceus | PV-1 | HM107293 |
| Sebastes rosaceus | BS-4 | GQ396750 |
| Sebastes rosaceus | AB-B-Cox1 | FJ158549 |
| Sebastes rosaceus | BG-2 | GQ501106 |
| Sebastes rosaceus | RA-1 | HM622583 |
| Sebastes rosaceus | RL-1 | GQ355774 |
| Sebastes rosaceus | NH-19 | JX295828 |
| Sebastes rosaceus | NH-7 | JX295816 |
| Sebastes rosaceus | NH-24 | JX295830 |
| Sebastes rosaceus | NH-8 | JX295814 |
| Sebastes rosaceus | NH-9 | JX295818 |
| Sebastes rosaceus | NH-10 | JX295813 |
| Sebastes rosaceus | NH-27 | JX295832 |

| | | |
|---|---|---|
| Sebastes rosaceus | NH-25 | JX295833 |
| Sebastes rosaceus | CO-2 | JX487242 |
| Sebastes rosaceus | NH-28 | JX295834 |
| Sebastes rosaceus | CO-3 | JX487245 |
| Sebastes rosaceus | CO-4 | JX487250 |
| Sebastes rosaceus | CO-5 | JX487253 |
| Sebastes rosaceus | CO-6 | JX487254 |
| Sebastes rosaceus | CO-7 | JX487256 |
| Sebastes rosaceus | NH-20 | JX295829 |
| Sebastes rosaceus | NH-23 | JX295827 |
| Sebastes rosaceus | RE-11 | JQ672546 |
| Sebastes rosaceus | NH-17 | JX295821 |
| Sebastes rosaceus | NH-26 | JX295835 |
| Sebastes rosaceus | NH-36 | JX295837 |
| Sebastes rosaceus | FT-3 | JX487258 |
| Sebastes rosaceus | CO-1 | JX487239 |
| Sebastes rubrivinctus | LO-13 | JX050981 |
| Sebastes rubrivinctus | CMB-4 | HQ231377 |
| Sebastes rubrivinctus | LO-16 | JX050984 |
| Sebastes rubrivinctus | LO-17 | JX050985 |
| Sebastes rubrivinctus | LO-10 | JX050978 |
| Sebastes rubrivinctus | FT-59 | JX110815 |
| Sebastes rubrivinctus | LO-18 | JX050986 |
| Sebastes rubrivinctus | LO-20 | JX487232 |
| Sebastes rubrivinctus | LO-12 | JX050980 |
| Sebastes rubrivinctus | BS-16 | GQ396762 |
| Sebastes rubrivinctus | BS-20 | GQ396766 |
| Sebastes rubrivinctus | FKM-2 | HM107299 |
| Sebastes rubrivinctus | GSV-1 | HM107305 |
| Sebastes rubrivinctus | CMB-19 | HQ229643 |
| Sebastes rubrivinctus | PS-07 | GQ254310 |
| Sebastes rubrivinctus | LO-15 | JX050983 |
| Sebastes rubrivinctus | LO-14 | JX050982 |
| Sebastes rubrivinctus | BS-18 | GQ396765 |
| Sebastes rubrivinctus | LO-19 | JX050987 |
| Sebastes rubrivinctus | LO-11 | JX050979 |
| Sebastes rubrivinctus | BS-15 | GQ396761 |

| | | |
|---|---|---|
| Sebastes rufus | SV-11 | JX487208 |
| Sebastes rufus | SV-12 | JX487209 |
| Sebastes rufus | 4-BC | GQ254414 |
| Sebastes rufus | SV-13 | JX487210 |
| Sebastes rufus | SV-14 | JX487211 |
| Sebastes semicinctus | GT-3 | GQ355770 |
| Sebastes semicinctus | NH-83 | JX444762 |
| Sebastes semicinctus | AC-1 | HM107290 |
| Sebastes semicinctus | FT-64 | JX110798 |
| Sebastes semicinctus | FT-67 | JX110800 |
| Sebastes semicinctus | FT-66 | JX110789 |
| Sebastes semicinctus | NH-96 | JX495620 |
| Sebastes semicinctus | AB-C-Cox1 | FJ158550 |
| Sebastes semicinctus | FT-70 | JX487223 |
| Sebastes semicinctus | FT-73 | JX487226 |
| Sebastes semicinctus | NH-70 | JX438529 |
| Sebastes semicinctus | AJ-2 | GQ355760 |
| Sebastes semicinctus | FT-54 | JX487218 |
| Sebastes semicinctus | CMB-11 | HQ231392 |
| Sebastes semicinctus | FT-56 | JX487220 |
| Sebastes semicinctus | CMB-8 | HQ231395 |
| Sebastes semicinctus | CMB-15 | HQ231390 |
| Sebastes semicinctus | CB-2 | HM622588 |
| Sebastes semicinctus | BD-1 | HM622585 |
| Sebastes semicinctus | NH-64 | JX438520 |
| Sebastes semicinctus | NH-57 | JX438516 |
| Sebastes semicinctus | NH-63 | JX438523 |
| Sebastes semicinctus | NH-62 | JX438524 |
| Sebastes semicinctus | NH-68 | JX438526 |
| Sebastes serranoides | AJ-4 | GQ355761 |
| Sebastes serranoides | SESE-08 | JN600319 |
| Sebastes serranoides | SESE-09 | JN600318 |
| Sebastes serranoides | SESE-10 | JN600317 |
| Sebastes serranoides | SESE-03 | JN582174 |
| Sebastes serranoides | SESE-04 | JN582173 |
| Sebastes serranoides | SESE-05 | JN582152 |
| Sebastes serranoides | SESE-06 | JN582172 |

| | | |
|---|---|---|
| Sebastes serranoides | VH-24 | JQ694030 |
| Sebastes serranoides | VH-36 | JQ694040 |
| Sebastes serranoides | NH-2 | JX295811 |
| Sebastes serranoides | SESE-07 | JN582171 |
| Sebastes serranoides | SESE-01 | JN582141 |
| Sebastes serranoides | SESE-02 | JN582140 |
| Sebastes serriceps | HA-7 | FJ501254 |
| Sebastes serriceps | CN-5 | FJ501253 |
| Sebastes serriceps | HA-6 | FJ501255 |
| Sebastes serriceps | AAE-2 | HQ229649 |
| Sebastes serriceps | HA-4 | FJ501256 |
| Sebastes serriceps | HA-3 | FJ501257 |
| Sebastes serriceps | AM-1 | HM107294 |
| Sebastes serriceps | AF-1 | HM107295 |
| Sebastes serriceps | BG-7 | GQ501101 |
| Sebastes serriceps | DD-8 | GQ254297 |
| Sebastes umbrosus | BG-6 | GQ501102 |
| Sebastes umbrosus | BG-5 | GQ501103 |
| Sebastes umbrosus | RS-3 | GQ501099 |
| Sebastes umbrosus | SZ-2 | HM107291 |
| Sebastes umbrosus | CB-5 | HM622584 |
| Sebastes umbrosus | BD-2 | HM622586 |
| Sebastes umbrosus | AB-E-Cox1 | FJ158551 |
| Sebastes umbrosus | NH-5 | JX295810 |
| Sebastes umbrosus | NH-16 | JX295823 |
| Sebastes umbrosus | BS-3 | GQ396751 |
| Sebastolobus altivelis | SEAL-8 | JX110786 |
| Sebastolobus altivelis | SEAL-1 | JX110782 |
| Sebastolobus altivelis | SEAL-2 | JX110794 |
| Sebastolobus altivelis | SEAL-6 | JX110785 |
| Sebastolobus altivelis | SEAL-5 | JX110795 |
| Sebastolobus altivelis | SEAL-9 | JX487235 |
| Sebastolobus altivelis | SEAL-3 | JX110783 |
| Sebastolobus altivelis | SEAL-4 | JX110784 |
| Sebastolobus altivelis | SEAL-7 | JX110796 |
| Sebastolobus altivelis | SEAL-10 | JX487236 |
| Semicossyphus pulcher | SEPU-03 | JQ934980 |

| | | |
|---|---|---|
| Semicossyphus pulcher | SEPU-02 | JQ934978 |
| Semicossyphus pulcher | SEPU-07 | JQ934967 |
| Semicossyphus pulcher | SEPU-09 | JQ934970 |
| Semicossyphus pulcher | SEPU-04 | JQ934979 |
| Semicossyphus pulcher | SEPU-01 | JQ934977 |
| Semicossyphus pulcher | SEPU-08 | JQ934968 |
| Semicossyphus pulcher | SEPU-10 | JQ934969 |
| Stylaster californicus | SC-9 | HQ603190 |
| Stylaster californicus | SC-13 | HQ603194 |
| Stylaster californicus | SC-14 | HQ603195 |
| Stylaster californicus | SC-16 | HQ603196 |
| Stylaster californicus | SC-17 | HQ603197 |
| Stylaster californicus | SC-11 | HQ603192 |
| Stylaster californicus | SC-12 | HQ603193 |
| Stylaster californicus | SC-10 | HQ603191 |
